# Manual therapy and exercise for patients with cluster headache

**DOI:** 10.17179/excli2021-3763

**Published:** 2021-05-25

**Authors:** Lucía de-la-Puente-Ranea, Alfonso Gil-Martínez, Oscar Rodríguez-Lopez, Pablo González-Gutiérrez, Maria Ángeles Mangas-Guijarro, Gonzalo Navarro-Fernández

**Affiliations:** 1 Departamento de Fisioterapia, Centro Superior de Estudios Universitarios La Salle, Universidad Autónoma de Madrid, Madrid, Spain; 2 Instituto de Rehabilitación Funcional, Centro Superior de Estudios Universitarios La Salle, Madrid, Spain; 3 CranioSPain Research Group, Centro Superior de Estudios Universitarios La Salle, Madrid, Spain; 4 Unidad de Fisioterapia, Hospital Universitario La Paz (IdiPAZ), Madrid, Spain; 5 Departamento de Fisioterapia, CEU San Pablo, Madrid, Spain; 6 Servicio de Neurología, Hospital Universitario La Paz, Madrid, Spain

**Keywords:** disability, manual therapy, exercise, cluster headache, pain, physiotherapy

## Abstract

The aim of this case series is to clarify if a physiotherapy program can reduce the frequency, intensity and duration of the headache episodes in patients with cluster headache. A 7-case series with cluster headache patients was conducted. Every subject received physiotherapy treatment based on manual therapy and exercise, maintaining pharmacological treatment prescribed by the neurologist. Frequency, intensity and duration of the episodes, pressure pain thresholds (PPT) and psychological variables were measured 5 times: pre-intervention, post-intervention, 1 month follow-up, 2 months follow-up and 3 months follow-up. Two of the seven subjects decreased their frequency of headaches over 50 % and another in 16,67 %. There were no significant changes in duration or intensity. Results also showed an improvement in impact of headache in 6 of 7 cases. Those cases that decreased more their headache frequency also decreased their pain catastrophizing. A program of physiotherapy based in manual therapy and exercise, might be an effective and safe complement to decrease the frequency of the episodes of CH in short-term (4 months follow-up) including interdisciplinary working with neurologists and other health care professionals.

## Abbreviations

CH Cluster Headache

CCH Chronic Cluster Headache

ECH Episodic Cluster Headache

PPT Pressure Pain Threshold

HIT6 Headache Impact Test

PCS Pain Catastrophizing Scale

PA Postero-Anterior

C2 Second Cervical Vertebra

V1 First Trigeminal Branch

V2 Second Trigeminal Branch

## Introduction

Cluster headache (CH) is the most disabling type of primary headache and it has been defined as a trigeminal-autonomous headache subtype by the International Classification of Headache Disorders, characterized by the onset of high intensity and short duration episodes of unilateral pain associated with autonomic symptoms (Halker et al., 2010[12]). Pain usually comprises the orbital, supraorbital and temporal areas, starting frequently in the upper jaw (May, 2013). In terms of frequency, CH presents both chronic (CCH) and episodic forms (ECH) (Chaibi and Russell, 2014[5]) and it is more usual in men (3:1), with an age of onset between twenty and forty years old (Weaver-Agostoni, 2013[34]). 

Based on clinical features of CH, it has been proposed in some studies that CH patients could have signs and symptoms suggesting a central sensitization process. It has been showed that CH patients have hyperalgesia (Bono et al., 1996[3]; Ladda et al., 2006[17]; Fernández-de-Las-Peñas et al., 2011[9]), allodynia (Marmura et al., 2009[20]), high risk of depression (Liang et al., 2013[19]) and decreased health-related quality of life even when appropriate treatments are used (D'Amico et al., 2002[7]). In 2019, Gil-Martínez et al. published a study determining that CH patients showed cranial and extracranial hyperalgesia, high levels of impact of headache, higher levels of anxiety and depression and lower levels of quality of life when compared with healthy subjects. Even more interesting is the fact that anxiety and depression were positively correlated with duration and frequency of headache, respectively (Gil-Martínez et al., 2019[11]).

Regarding this complex clinical situation and the burden of the disease (Jensen et al. 2007[14]), some pharmacological and non-pharmacological treatments have been proposed in order to reduce the impact of the headache in patient's lives. Among pharmacological options, verapamil, topiramate and litium stand out as a preventive treatment and oxygen 100 %, subcutaneus sumatriptan, oral corticosteroids and occipital nerve block as abortive treatment (Becker, 2013[1]).

Furthermore, other conservative treatment frequently used in the management of primary headache patients is physical therapy and therapeutic exercise. However, although the efficacy of physiotherapy has been studied for other primary headaches (Chaibi et al., 2011[6]), there are no studies evaluating the effects of physiotherapy in patients with CH controlled with basal medication. There is only one study published in relation to physical therapy on CH, a case report in which manual therapy and therapeutic exercise, combined with neurostimulation and medication, seems to be useful in the treatment of CH. Regarding all primary headaches, some of the techniques which have demonstrated hypoalgesic effects are manual therapy (Chaibi and Russell, 2014[5]) and therapeutic exercise (Gil-Martínez et al., 2013[10]). Specifically, it appears that the upper neck region may be an important target for physiotherapeutic approaches in CH patients. Recently, an article was published showing promising results in occipital nerve stimulation (Díaz-de-Terán et al., 2021[8]). 

Therefore, the aim of this case series is to clarify if a physiotherapy program based on manual therapy and therapeutic exercise can reduce the frequency, intensity and duration of the headache episodes in patients with CH.

## Methods

This study was carried out in a private physiotherapy center and in a tertiary public hospital. The study had a total duration of 8 months.

### Participants and evaluation description

Cases were adults aged 18 to 65 years who had been diagnosed with CCH or ECH by a neurologist. All patients signed the informed consent before the beginning of the study. All subjects with systemic diseases, fibromyalgia, peripheral neuropathies, craneomandibular dysfunctions and recent trauma or chirurgic interventions in neck or facial areas were excluded.

Five evaluations were included in the case series: pre-intervention, post-intervention and one, two and three months after the post-intervention. In the first evaluation, the subjects who accepted the participation in the study were informed about the study by a physiotherapist. After that, a personal interview was carried out to obtain information about the pharmacological treatment, demographical data and specific characteristics of pain, such as frequency, intensity and duration of the headache. 

The pain pressure threshold (PPT) of facial and peripheral areas was measured in every evaluation using a digital algometer (Fx.25 Force Gage model, Wagner Instruments, Greenwich, CT, USA). The PPT bilateral measurements were: the two upper branches of the trigeminus nerve (V1, in supraorbital keyhole and V2, in infraorbital keyhole) and tibialis anterior. To conclude, each subject filled the Headache Impact Test-6 (HIT6) (Sauro et al., 2010[28]), the Pain Catastrophizing Scale (PCS) (Olmedilla Zafra et al., 2013[24]) and the disability and daily life affection were measured with a non-verbal scale where 0 was no affection and 10 the greatest affection. 

### Treatment

**Figure 1:**
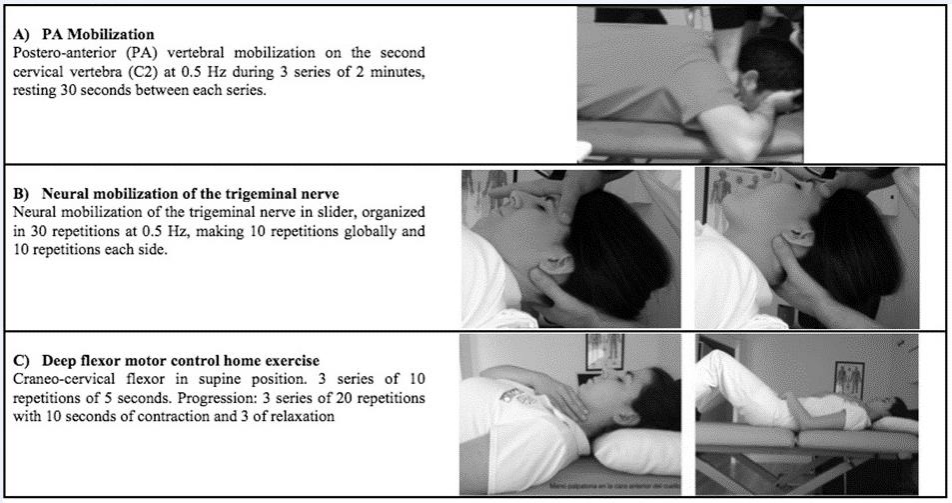
Physiotherapy program

A physiotherapy program was applied, without making any changes in basal medication prescribed by the neurologist. Eight sessions were carried out; organized in 2 sessions per week for 1 month. The first technique was a postero-anterior (PA) vertebral mobilization on the second cervical vertebra (C2) at 0.5 Hz during 3 series of 2 minutes, resting 30 seconds between each series (La Touche et al., 2013[16]). The second technique was a neural mobilization of the trigeminal nerve, organized in 30 repetitions at 0.5 Hz, making 10 repetitions globally and 10 repetitions each side, increasing the tension in the auriculotemporal nerve area. After that deep flexor motor control home exercises were prescribed as described by Harris et al. (2005[13]) (Figure 1(Fig. 1)).

## Results

Seven subjects were recruited (2 with CCH and 5 with ECH). Among the patients with ECH, 4 of them were in remission period (number 4 to number 7) and only 1 subject was in active period (case number 3). Demographic characteristics are described in Table 1(Tab. 1).

One of the two subjects with CCH decreased its headache frequency more than a 50 % of his basal frequency after treatment; and the other subject decreased his headache frequency a 16.67 %. Among the patients with ECH, only one of the cases in active period decreased the headache frequency in more than a 50 %. There were no changes in ECH in remission period (Table 2(Tab. 2)).

Concerning PPT, an increase in V1 mean from pre-treatment to 3^rd^ month follow-up was observed, from 0.53 (0.21) to 0.63 (0.27). A decrease was also reported in HIT-6 mean from pre-treatment to 3^rd^ month follow-up, from 60.43 (8.85) to 50.57 (8.16), and in PCS from 32.86 (3.17) to 21.14 (18.18) (Tables 3(Tab. 3) and 4(Tab. 4)).

Disability and daily life affection were also decreased in patients with CCH, and a complete disability reduction was reported in the subject with ECH in active period, maintaining with no differences in subjects with ECH in remission period. 

## Discussion

According to clinical guidelines in CH, there are differences between patients with CCH and ECH (Lademann et al., 2015[18]). Nevertheless, in this case series patients from both categories have been included to approach the epidemiology of both entities. 

Considering the obtained data, a decrease in frequency of headaches was reported in subjects with both CCH and ECH in active period. These results may have been caused by the analgesic effect generated by the physiotherapy techniques, which in addition to the pharmacological treatment may have this effect. Also, the results obtained in this case series were similar to those obtained in another previous study (Navarro-Fernández et al., 2019[23]). 

Joint mobilization has demonstrated an analgesic effect in other types of headache (Chaibi et al., 2011[6]; La Touche et al., 2013[16]), activating descending pain pathways such as noradrenergic and serotoninergic pathways, which involve both supraspinal and grey periaqueductal substance areas (Skyba et al., 2003[29]). 

Furthermore, it has been reported that neural mobilization produced a normalization in the expression of astrocytes and microglial cells in the posterior horn (Martins et al., 2011[21]; Santos et al., 2012[27]). Thus, neural mobilization could decrease glial activity, which is implied in chronification of pain.

Likewise, it has been observed that a neck and cranium motor control training can reduce frequency, intensity and duration of headaches in the long term (Busch and Gaul, 2008[4]). This could be due to an activation in opioid pathways after exercising, which triggers a liberation of β-endorphins in hypothalamus and grey periaqueductal substance areas, activating pain inhibition pathways. Moreover, there are reviews which support a treatment based in therapeutic exercise with cardiovascular exercise in primary headaches (Gil-Martínez et al., 2013[10]), even in a preventive treatment (Varkey et al., 2011[31]). 

No moderate changes in the intensity, duration, and number of episodes of CH were observed in subjects in the active period; hence, the need for future studies with a larger sample size.

Regarding PPT, an increase in the mean of V1 when comparing pre-intervention and 3 months follow-up and a decrease in V2 and tibialis anterior was reported. These last results are contrary to previous studies, in which the combination of the applied techniques in the cervical segment triggered an increase of the PPT in facial areas (La Touche et al., 2009[15]).

Nevertheless, the reported changes in tibialis anterior do not reach the minimal detectable change (Walton et al., 2011[33]), which indicates that the sample size should be increased so that the effects of the physiotherapy techniques on PPT in CH are observed.

With reference to psychological factors, 6 out of 7 cases improved the daily life affection of the CH. This may be a future line of research, as high results in this variable have been reported previously both in CCH and ECH in active or remission period (Torkamani et al., 2015[30]).

The obtained results indicate that those cases with a higher rate of episodes decrease more their catastrophism. This may indicate a relationship between pain catastrophizing and other headache-related variables such as frequency, chronicity, duration, self-sufficiency (Bond et al., 2015[2]), disability and pain intensity (Queralto, 2005[25]). Such relationship has been already proved in the classic model of Vlaeyen and Linton (2000[32]).

In a similar way, subjects who decreased their rate of episodes also decreased their disability. Similar results were reported by Ragi et al. in a study in which the direct contact between patient and professional triggered a reduction in disability in patients with migraine (Raggi et al., 2012[26]). No adverse effects were reported.

### Implications for physiotherapy practice

The results obtained in this case series seem to be supportive of the idea that physical therapy interventions based on exercise and manual therapy could be beneficial for cluster headache patients. However, considering that this is a case series, these preliminary results should be interpreted with caution. 

Exercise and manual therapy reduced disability and daily live affection of cluster headache patients in this case series. 

Physical therapy interventions based on manual therapy and exercise seemed to improve physical and psychological variables of cluster headache patients.

### Limitations

There are many limitations to consider in this case series. First, the results cannot be extrapolated to the rest of population. Nevertheless, the investigators consider that this case of series is a preliminary tool to further investigations. Second, the data interpretation should be conservative, due to the study design and the lack of a control group. Third, the consumption of analgesic drugs may have influenced in the patient condition. It should also be considered that the recruitment was partially carried out in an Association of CH, which might imply common expectation on the treatment. The lack of literature related with CH indicates a need to carry out more investigations that observe the effect of physiotherapy on CH, making a separated classification of CCH, ECH in both active and remission period. Last, the lack of previous studies in which PPTs minimum detectable change and clinically relevant change were obtained in the facial area.

In conclusion, a program of physiotherapy based in manual therapy and exercise, might be an effective and safe complement to decrease the frequency of the episodes of CH in short-term (4 months follow-up) including interdisciplinary working with neurologists and other health care professionals.

## Conflict of interest

No potential conflict of interest relevant to this article was reported. This research did not receive any specific grant from funding agencies in the public, commercial, or not-for-profit sectors.

## Acknowledgements

The authors would like to sincerely thank the patients who graciously participated in this case of series. We also thank Centro de Estudios Superiores La Salle for its assistance with the English edition of the manuscript.

## Figures and Tables

**Table 1:**
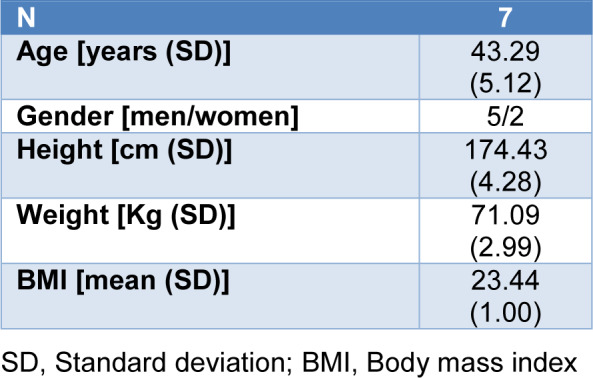
Demographic characteristics of the patients

**Table 2:**
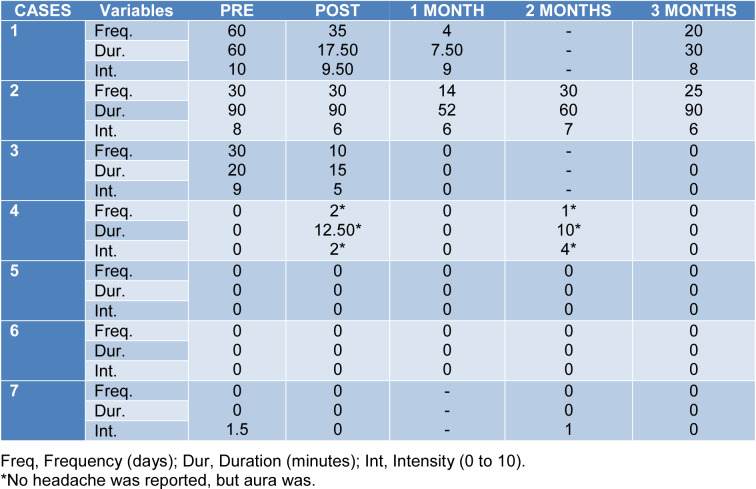
Subjective evolution of pain per case

**Table 3:**
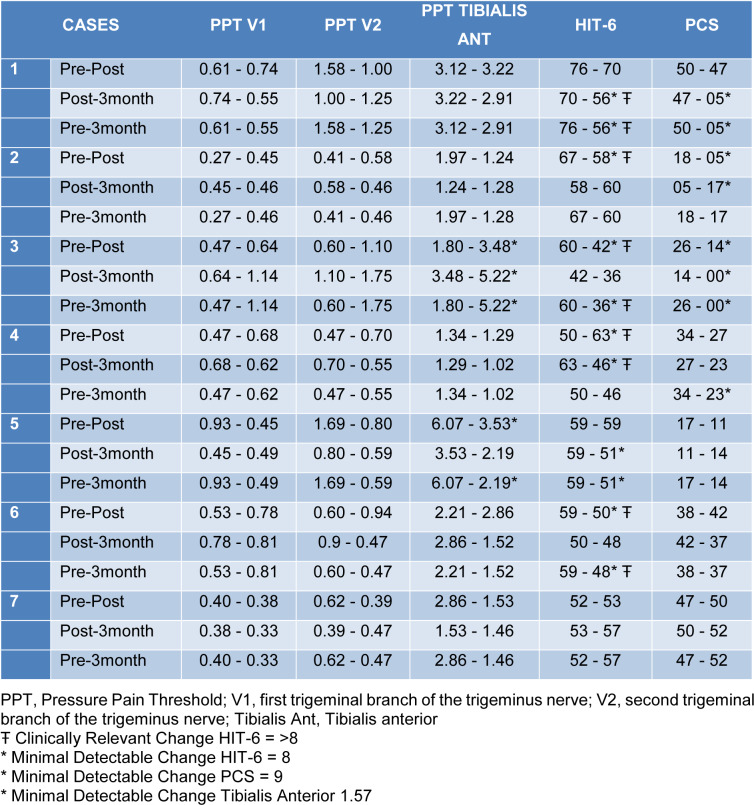
Somatosensorial characteristics of the affected side and psychological characteristics

**Table 4:**
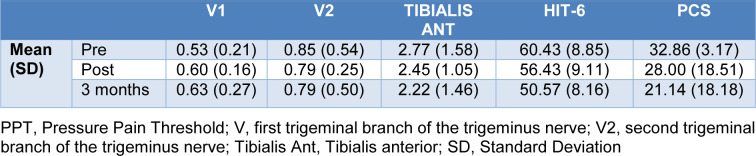
Means and Standard deviation of somatosensorial characteristics of the affected side and psychological characteristics (all cases)
